# Evaluating eight smoking metrics for modelling survival in non-small cell lung cancer

**DOI:** 10.1016/j.canep.2026.103052

**Published:** 2026-03-20

**Authors:** Andrew CL Lam, Yao Li, M.Catherine Brown, Yangqing Deng, Katrina Hueniken, Natasha B. Leighl, Frances A. Shepherd, Kiera Murison, Zhichao Wang, Jui Kothari, Angela S. Wenzlaff, Haoran Liu, Takashi Kohno, Angela Cecilia Pesatori, Curtis Harris, Hongxia Ma, Juncheng Dai, Matthew J. Barnett, Ryan Diver, Leticia Ferro Leal, Guillermo Fernandez-Tardon, Mónica Pérez-Ríos, Michael PA Davies, Bernd Holleczek, Paul Brennan, David Zaridze, Ivana Holcatova, Jolanta Lissowska, Beata Świątkowska, Dana Mates, Milan Savic, Hermann Brenner, Angeline S. Andrew, Fiona Taylor, John K. Field, Alberto Ruano-Ravina, Sanjay S. Shete, Adonina Tardon, Ying Wang, Loic Le Marchand, Rui Manuel Reis, Matthew B. Schabath, Marian L. Neuhouser, Hongbing Shen, Maria Teresa Landi, Kouya Shiraishi, Jie Zhang, Ann G. Schwartz, Ming S. Tsao, David C. Christiani, Ping Yang, Rayjean J. Hung, Wei Xu, Geoffrey Liu

**Affiliations:** a Department of Medicine, University of Toronto, Toronto, Ontario, Canada; b Dalla Lana School of Public Health, University of Toronto, Ontario, Canada; c The Princess Margaret Cancer Centre, University Health Network, Toronto, Ontario, Canada; d Department of Biostatistics, University Health Network, Toronto, Ontario, Canada; e Lunenfeld-Tanenbaum Research Institute, Sinai Health Systems, Toronto, ON, Canada; f Department of Quantitative Health Sciences, Mayo Clinic, Scottsdale, AZ, USA; g Division of Pulmonary and Critical Care Medicine, Jiangsu Province Hospital of Chinese Medicine, Nanjing University of Chinese Medicine, Nanjing, Jiangsu, China; h Harvard T H Chan School of Public Health, Harvard University, Boston, MA, USA; i Barbara Ann Karmanos Cancer Institute, Wayne State University, Detroit, MI, USA; j Department of Thoracic Surgery, Shanghai Chest Hospital, Shanghai Jiaotong University, China; k Division of Genome Biology, National Cancer Center Research Institute, Tokyo, Japan; l Department of Clinical Sciences and Community Health, University of Milan, Italy; m Centre for Cancer Research, National Institutes of Health, Bethesda, MD, USA; n Department of Epidemiology, School of Public Health, Nanjing Medical University, Nanjing, China; o Cancer Prevention Program, Public Health Sciences, Fred Hutchinson Cancer Center, Seattle, WA, USA; p American Cancer Society, Atlanta, GA, USA; q Molecular Oncology Research Center, Barretos Cancer Hospital, Barretos, Brazil; r Barretos School of Health Sciences Dr. Paulo Prata - FACISB, Brazil; s Facultad de Medicina y CC Salud, Universidad Nebrija, Madrid, Spain; t Health Research Institute of Asturias (ISPA), Oviedo, Spain; u Department of Preventive Medicine and Public Health, University of Santiago de Compostela, Spain; v CIBER de Epidemiología y Salud Pública, CIBERESP, Spain; w Roy Castle Lung Cancer Research Programme, Department of Molecular and Clinical Cancer Medicine, University of Liverpool, Liverpool, UK; x Saarland Cancer Registry, Saarbrücken, Germany; y Genomic Epidemiology Branch, International Agency for Research on Cancer/World Health Organization, Lyon, France; z N.N. Blokhin National Medical Research Centre for Oncology, Moscow, Russia; aa Department of Oncology, 2nd Medical Faculty, Charles University and University Hospital Motol, Institute of Hygiene and Epidemiology, 1st Faculty of Medicine, Charles University, Prague, Czech Republic; ab M.Sklodowska-Curie National Research Institute of Oncology, Wawelska 15B, Warsaw 02-034, Poland; ac Department of Environmental Epidemiology Nofer Institute of Occupational Medicine, 8 Teresy St., Łódź 91-348, Poland; ad National Institute of Public Health, Bucharest, Romania; ae Department of Thoracic Surgery, Clinical Center of Serbia, Belgrade, Serbia; af Cancer Prevention Graduate School, German Cancer Research Center (DKFZ), Heidelberg, Germany; ag Dartmouth-Hitchcock Medical Center, Lebanon, NH, USA; ah Department of Oncology and Metabolism, University of Sheffield, and Weston Park Hospital, Sheffield, UK; ai University of Sheffield and Sheffield Teaching Hospitals Foundation Trust, Sheffield, UK; aj Health Research Institute of Santiago de Compostela (Instituto de Investigación Sanitaria de Santiago de Compostela-IDIS), Santiago de Compostela, Spain; ak M D Anderson Cancer Center, University of Texas, Houston, TX, USA; al University of Hawaii Cancer Centre, HI, USA; am ICVS/3B’s-PT Government Associate Laboratory, Braga, Portugal; an Life and Health Sciences Research Institute (ICVS), Medical School, University of Minho, Braga, Portugal; ao H Lee Moffitt Cancer Center and Research Institute, Tampa, FL, USA; ap National Cancer Institute, National Institutes of Health, Bethesda, MD, USA; aq Department of Clinical Genomics, National Cancer Center Research Institute, Tokyo, Japan

**Keywords:** Lung cancer, Overall survival, Cancer survival, Cigarette smoking, Smoking metric

## Abstract

**Introduction:**

Smoking is a strong modifiable prognostic factor for lung cancer survival. We compared eight smoking metrics to determine which metric best models the relationship between smoking exposure with overall survival (OS) and lung cancer-specific survival (LCSS). These metrics included cigarettes-per-day, smoking duration, pack-years, square-root pack-years, logcig-years, the comprehensive smoking index, age-of-initiation, and years-since-quit.

**Materials/methods:**

This retrospective, pooled analysis included 25 International Lung Cancer Consortium studies between June 1, 1983–December 31, 2019. The performance of smoking metrics for modelling OS was compared based on 1) strength and significance in adjusted Cox-proportional hazard models and 2) linearity based on the goodness-of-fit assuming the log-hazard varies linearly with each smoking metric (i.e. the hazard ratio is constant across different values of the smoking metric) compared to models using non-linear splines. This process was repeated across clinicodemographic subgroups and for LCSS.

**Results:**

In total, 28,702 lung cancer patients were included (median age 64 [IQR: 57–71]; 53% male). Logcig-years (log(cigarettes/day+1)⋅years-smoked) had the highest adjusted hazard ratio per standard deviation (aHR 1.11; 95% CI: 1.09–1.13) and best goodness-of-fit when modelled linearly. Square-root pack-years had a similar effect size (aHR 1.11; 95% CI: 1.09–1.13) and had a strong linear relationship on visual assessment of spline curves. In subgroup analyses, logcig-years had a large effect size and maintained a linear relationship regardless of age, sex, stage, and histology. For lung cancer–specific survival (LCSS), logcig-years again had the highest aHR (1.09; 95% CI: 1.05–1.12) and the best linear goodness-of-fit, while square-root pack-years demonstrated the most linear relationship on visual assessment.

**Discussion:**

Logcig-years best modelled the relationship between smoking exposure and OS as well as LCSS, and had consistent associations across clinicodemographic subgroups. Logcig-years should be considered in clinical and research applications for quantifying smoking exposure in lung cancer.

## 1. Introduction

Lung cancer (LC) is the most commonly diagnosed cancer and the leading cause of cancer-related mortality worldwide [1]. Cigarette smoking is not only a strong risk factor for developing LC but also a strong prognostic factor after diagnosis [2–5]. For example, in a cohort of 26,957 patients, Kawaguchi et al. reported that never-smokers lived an average of 11 months longer than ever-smokers after controlling for other prognostic factors (30 vs. 19 months; p < 0.001) [4]. This study highlights the importance of accurately characterizing smoking exposure when studying survival outcomes in LC.

In clinical practice and research, smoking exposure is commonly characterized as binary smoking status (never vs. ever-smoker) or as pack-years. While easy to document, binary smoking status does not capture the cumulative dose-response relationship between smoking and survival [6]. Conversely, pack-years is a continuous smoking metric widely-used in clinical practice and research since the 1950s [7,8]. However, pack-years assumes a linear relationship and assigns equal weight to smoking dose and duration despite evidence suggesting that the underlying dose–response relationship may be nonlinear and duration may be more strongly associated with survival [9,10]. Furthermore, apart from dose and duration, there are other dimensions of smoking exposure that are independent prognostic factors for survival. These include age-of-initiation [11], time-to-first cigarette [12], and smoking abstinence duration [5].

To address these limitations, several alternative smoking exposure metrics have been proposed. Some apply mathematical transformations to smoking exposure to better model potentially nonlinear dose–response relationships. For instance, logcig-years applies a logarithmic transformation to smoking intensity and was developed by Thurston et al. based on the concept that carcinogenic binding to DNA may follow a logarithmic relationship [13]. Similarly, square-root pack-years attenuates the influence of high smoking exposure and models the plateauing effects of smoking exposure on LC survival [10]. Other metrics incorporate additional dimensions of smoking exposure not captured by conventional metrics. For example, the comprehensive smoking index (CSI) was proposed by Dietrich and Hoffman and incorporates smoking dose, duration, and time-since-cessation into a single metric [14,15]. While many of these metrics were developed and validated for LC risk prediction, where they often demonstrated improved performance compared to pack-years, their ability to model survival has not been systematically evaluated.

To our knowledge, no studies have evaluated multiple smoking metrics in a head-to-head comparison for modelling survival within a LC cohort [16,17]. In this study, we leveraged large-sample data from the International Lung Cancer Consortium (ILCCO) to compare the performance of eight smoking metrics for modelling the association between smoking exposure with overall survival (OS) and LC-specific survival (LCSS) in patients with non-small cell lung cancer (NSCLC), which accounts for ~85% of all LC diagnoses [18]. The goal of this study is to determine which smoking metric best captures the relationship between exposure and survival, and whether alternative metrics may supplant the conventionally used pack-years metric.

## 2. Methods

### 2.1. Study design and participants

Patient-level data were pooled across 25 studies participating in ILCCO from 22 sites spanning 10 countries. Data were collected between June 1, 1983–December 31, 2019. Data analysis was performed between May 1, 2022 and June 30, 2025. Ethics approval was obtained from each local ethics board. Details of the ILCCO consortium, including inclusion/exclusion criteria, are described on the consortium website [19] and in previous studies [5,20]. See Supplementary material 1 for details regarding participant details, metric collection, and data harmonization/management.

### 2.2. Smoking metrics

Eight continuous smoking metrics were included in this analysis. Base smoking metrics, including cigarettes-per-day, age-of-initiating smoking, and smoking abstinence duration were self-reported by patients at the time of study enrolment. Smoking duration was either self-reported or calculated based on self-reported start and stop age. Calculated smoking metrics, including pack-years, square-root pack-years, logcig-years, and the comprehensive smoking index (CSI) were computed based on base smoking metrics [13,15]. Formulas for calculated smoking metrics were shown in Supplementary material 2.

For never-smokers, cigarettes-per-day, duration, pack-years, logcig-years, and CSI were all set to a value of zero. Smoking abstinence duration and age-of-initiating smoking could not be calculated in this population. Thus, never-smokers were excluded from models involving smoking abstinence duration and age-of-initiating smoking. Patients were considered former-smokers only if they were abstinent for greater than two years from date-of-diagnosis. This definition accounts for transient smoking cessation that often occurs immediately after a cancer diagnosis [21].

### 2.3. Covariates

Covariates included in this analysis were age-at-diagnosis, sex, race, education, body mass index (BMI), cancer stage-at-diagnosis (using the American Joint Commission on Cancer TNM staging 7th edition and included in models as three categories: I, II/III, and IV), histology, and year-of-diagnosis. These variables were selected a priori based on previously established prognostic factors for NSCLC survival and their availability within the consortium database [22]. These covariates were included in adjusted models to control for potential confounding in the association between smoking exposure and survival outcomes.

### 2.4. Study aims and statistical analysis

#### 2.4.1. Aim 1: modelling overall survival

The primary outcome was OS, which was measured in years from diagnosis until the date of last follow-up or any-cause death. We generated unadjusted Kaplan-Meier (KM) curves, grouped by quartiles for each smoking metric, to visualize the relationship between each metric and OS.

The performances of eight smoking metrics were judged based on three criteria:

1. strength of association with survival (as determined by the adjusted Cox-proportional hazards models)
2. model goodness-of-fit (as determined by the Akaike Information Criteria (AIC)-linear value)
3. linearity of the relationship between smoking metrics and the log-hazard; or how well the information from a given smoking metric is captured by a single, constant hazard ratio when modelling overall survival (as determined based on goodness-of-fit testing and visual examination of model fit).

To examine the association between smoking metrics and OS (criterion #1) as well as AIC-linear (#2), we generated both unadjusted and adjusted Cox proportional-hazards regression models. Unadjusted models were still adjusted for smoking status as most smoking metrics did not incorporate smoking status. Adjusted models included covariates that are established prognostic factors for NSCLC. Hazard ratios (HR) and AIC-linear were compared across smoking metrics from the adjusted models. The above Cox models used a single HR coefficient to describe each continuous smoking metric [23]. This model assumes a linear relationship between a given smoking metric and the log-hazard of any-cause death. A lower AIC-linear indicates a better model fit under the assumption of a linear relationship with log hazard.

Our goal was to identify metrics with strong linear relationships to the log-hazard that do not require complex modelling strategies to capture. To do this, a second Cox model was fitted, relaxing this assumption of linearity by using penalized splines with 2–5 knots, allowing the hazard ratio to vary across different values of the smoking metric [24]. The best number of knots was selected based on the Akaike Information Criterion (AIC). We defined “p-linear” as the Wald test p-value for the regression coefficient of each smoking metric from our first model, assuming a linear effect on the log-hazard. “P-nonlinear” was defined as the p-value of a likelihood ratio test between our spline model and the model assuming linearity, to determine whether allowing a non-linear effect on the log-hazard significantly improved model fit.

The ideal smoking metric has a strong and significant relationship with OS based Cox-proportional hazards model. It also has the lowest AIC-linear, indicating the best model fit [23]. Finally, it should have a significant p-linear, non-significant p-nonlinear, and linear relationship on visual assessment of its spline; this indicates a linear relationship with OS. A linear relationship between smoking metrics and survival is preferred due to its simplicity, ease of interpretation, lower risk of overfitting, and greater statistical efficiency [25]. Modelling log-hazard ratios linearly across a continuous variable also typically yields more stable estimates, as they involve fewer degrees-of-freedom and tend to have more statistical power compared to more flexible models, which have higher degrees of freedom.

#### 2.4.2. Aim 2: modelling overall survival in clinicodemographic subgroups

We conducted subgroup analyses by stratifying patients based on clinicodemographic factors: age (<65 vs. ≥65), sex (male vs. female), smoking status (current- vs. former-smokers), cancer stage (non-metastatic defined as stages I–III vs. metastatic defined as stage IV), and histology (adenocarcinoma vs. squamous). An exploratory analysis was conducted to determine metric performance within individual cancer stages. The same criteria used to measure metric performance in Aim 1 were applied to this aim.

#### 2.4.3. Aim 3: modelling lung cancer-specific survival

Finally, we compared the eight smoking metrics for modelling LCSS. The same criteria used to determine the optimal smoking metric in Aim 1 were again applied for LCSS. Cause-specific hazard ratios were computed via Cox regression. All analyses were conducted with R v4.4.1 [26]. All tests of significance were two-sided; analyses were considered statistically significant at p < 0.05.

## 3. Results

### 3.1. Study population

In total, 35,396 patients from the ILCCO dataset were assessed for eligibility and 28,702 had complete data to be included for analysis. Fig. S1 illustrates the Consolidated Standards of Reporting Trials (CONSORT) diagram for this analysis. Clinicodemographic and smoking metrics for the final study population are presented in Table 1. Of the analyzed patients (n = 28,702), 7167 were never-smokers, 12,883 were current-smokers, and 8652 were former-smokers. Detailed information on the study population stratified by study site, including study characteristics, is presented in Table S1.

### 3.2. Aim 1: modelling overall survival

Unadjusted KM curves were constructed to illustrate the association between varying intensities of each smoking metric and OS (Fig. S2). These curves demonstrate that increased smoking exposure and shorter smoking abstinence durations were associated with reduced OS.

**Fig. 1.**
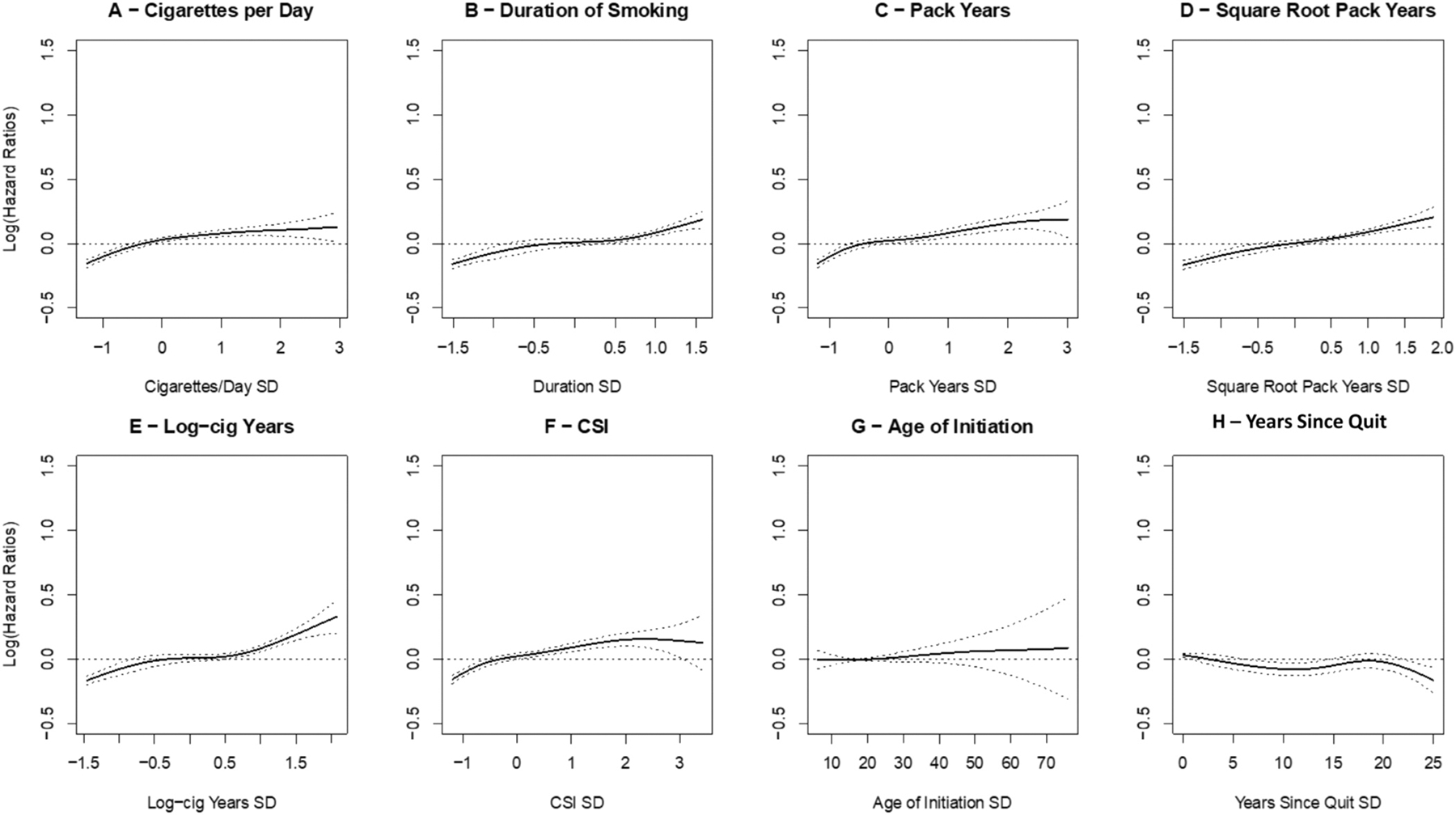
Spline-curves for eight smoking metrics in relation to overall survival adjusted for smoking status, age, sex, race, education, body mass index, cancer stage, histology, and year of diagnosis. Note that age of initiation and years since quit have a different sample size than the other smoking metrics. Abbreviations: CSI, comprehensive smoking index.

Table 2 outlines the adjusted Cox proportional-hazard models, AIC-linear, p-linear, and p-nonlinear for all eight smoking metrics; Table S2 outlines the unadjusted models. Logcig-years and square-root pack-years had the strongest relationship with OS in the adjusted Cox-proportional hazards model (aHR 1.11; 95% CI 1.09–1.13 for both). Of the two, logcig-years had the lowest AIC-linear and a significant linear relationship with OS (p-linear <0.001). Square-root pack-years also had a significant linear relationship (p-linear <0.001) and a non-significant nonlinear relationship (p = 0.15). Both metrics had a larger effect size and a better linear model fit compared to pack-years. Fig. 1 illustrates the splines for the eight smoking metrics. By visual assessment, square-root pack-years had the most linear relationship with OS.

### 3.3. Aim 2: modelling overall survival in clinicodemographic subgroups

Table 3 outlines the adjusted Cox proportional-hazard models, AIC-linear, p-linear, and p-nonlinear for each smoking metric stratified by age, sex, smoking status, cancer stage, and histology. Fig. S3 illustrates the splines between each metric and OS, also stratified by clinic-demographic subgroups. Although all eight metrics were analyzed, for ease of presentation, only the three highest performing smoking metrics from Aim 1 (logcig-years, square-root pack-years, and duration) were included. In addition, pack-years was included as it is the conventional smoking metric used in clinical and research practice.

Regardless of age, logcig-years had the largest effect size and lowest AIC-linear of all smoking metrics. Logcig-years also had a significant p-linear (p < 0.001) and a non-significant p-nonlinear (p = 0.13 for <65; p = 0.57 for ≥65). On visual assessment of the splines, logcig-years had a linear relationship with OS. When stratified by sex, logcig-years had the highest aHR (1.11; 95% CI 1.08–1.14) and lowest AIC linear in men. It was also significantly linear (p < 0.001) with a non-significant p-nonlinear (p = 0.12). In females, both logcig-years and square-root pack-years had the highest aHR (1.10; 95% CI 1.07–1.13 for both) but square-root pack-years had a slightly lower AIC linear. On visual assessment, both logcig-years and square-root pack-years had a linear relationship with OS in men, while in females, the splines demonstrate a non-linear relationship across all four metrics.

Among current-smokers, square-root pack-years and pack-years both had the highest aHR (1.04; 95% CI 1.02–1.07 for both), lowest AIC-linear, and a significant p-linear (p = 0.002 for both); only pack-years had a non-significant p-nonlinear (p = 0.21). Among former-smokers, pack-years had the highest aHR (1.06; 95% CI 1.03–1.09) and lowest AIC. On visual assessment, none of the four smoking metrics had a strong relationship with OS regardless of smoking status. When stratified by cancer stage, logcig-years had the highest aHR (1.13; 95% CI 1.10–1.16), lowest AIC, and significant p-linear (p < 0.001) in non-metastatic patients, while square-root pack-years performed the best in metastatic disease with the highest aHR (1.09; 95% CI 1.06–1.13) and lowest AIC. This is congruent with visual assessment of the splines as both logcig-years and square-root pack-years had a linear spline when stratified by stage. We performed an exploratory analysis and stratified the cohort into individual stages. Logcig-years had the largest effect size, lowest AIC-linear, and the most linear spline on visual assessment in stages I and II. Square-root pack-years had the largest effect size and lowest AIC-linear in stages III and IV. See Table S3 and Fig. S4 for this exploratory analysis.

Finally, when stratified by histology, logcig-years had the highest aHR, lowest AIC, and a significant p-linear (p < 0.001 for adenocarcinoma; p = 0.005 for squamous cell) in both adenocarcinoma and squamous cell carcinoma patients. Visually, both logcig-years and square-root pack-years had linear splines regardless of histology.

### 3.4. Aim 3: modelling lung cancer-specific survival

Table 4 outlines the adjusted Cox proportional-hazard models, AIC-linear, p-linear, and p-nonlinear for the eight smoking metrics in relation to LCSS. Results of the unadjusted models for LCSS are presented in Table S2. Logcig-years had the highest aHR in the Cox-proportional models (1.09; 95% CI 1.05–1.12), the lowest AIC-linear, and a significant linear relationship with OS (p < 0.001). However, it also had a significant p-nonlinear relationship (p = 0.002). This is reflected in the splines as pack-years and square-root pack-years had a more linear spline curve on visual assessment. The spline curves for the eight smoking metrics are illustrated in Fig. S5.

## 4. Discussion

This study leveraged data from 28,702 NSCLC patients from ILCCO to compare eight smoking metrics for modelling smoking exposure with OS and LCSS. This study had three major findings. Firstly, logcig-years best modelled the relationship between smoking and OS. Square-root pack-years also had comparable performance and is a reasonable alternative when a strong linear relationship is needed. Both smoking metrics supplanted the conventionally used pack-years and other base metrics like cigarettes-per-day and smoking-duration. Secondly, logcig-years performed well across clinicodemographic subgroups including age, sex, stage, and histology. Finally, logcig-years best modelled the relationship between smoking exposure and LCSS.

Logcig-years was developed based off observations that DNA adducts (a driver of carcinogenesis from smoking) form through a logarithmic relationship with smoking exposure, and that the accumulation of DNA adducts correlates well with LC risk [13]. Therefore, a smoking exposure modeled as a logarithmic function should predict LC risk well. Mathematically, logcig-years places greater emphasis on smoking-duration compared to smoking dose (cigarettes-per-day). This is in keeping with previous studies that show smoking-duration to be a much stronger predictor of LC risk and mortality than smoking dose [9].

Square-root pack-years accounts for the “reduced potency” phenomenon, where the relative impact of smoking on LC risk and mortality diminishes with increasing exposure [27]. Mechanistically, this may be explained by saturation of metabolic pathways responsible for activating pro-carcinogens at high smoking exposures. Furthermore, behavioral studies suggest that heavy smokers may inhale less smoke per cigarette and leave larger remnants in cigarette butts compared to light smokers [28]. Consequently, modelling exposure as a square-root function attenuates the effects of heavy smoking on survival.

Previous studies have compared smoking metrics in the LC risk setting. For instance, Thurston et al. used a dataset of 1275 cases and 1269 controls to compare the performance of metrics (including pack-years, square-root pack-years, and logcig-years) for modelling LC risk [13]. Logcig-years and square-root pack-years modeled the exposure-risk relationship better than pack-years. Both had a significant, linear relationship unlike pack-years that had a highly nonlinear relationship. In a separate study by Remen et al., 1434 cases and 1513 controls were used to compare four continuous metrics (duration, intensity, pack-years, and CSI) for modelling LC risk [10]. CSI best modelled LC risk, while duration alone performed similarly if not better than pack-years. Several key points emerged from these studies. Firstly, there were smoking metrics available that modelled LC risk better than pack-years. Secondly, smoking-duration alone was a strong predictor of risk and often performs as well, if not better, than pack-years.

Our study extends these observations when modelling survival, confirming the limitations of pack-years persist when modelling exposure with survival. Our group performed a similar analysis of the same eight smoking metrics for modelling survival in head and neck cancer patients [29]. In that study, logcig-years also emerged as the highest performing metric with smoking-duration also performing well. Taken as a whole, this suggests that logcig-years is a robust metric for modelling the relationship between smoking exposure with cancer risk and survival, across two cancer sites.

Logcig-years also performed well in modelling exposure and LCSS, indicating that it not only captures the impact of smoking on OS but also isolates the impact on LCSS. This is important as NSCLC patients often present with a competing risk of noncancer-related mortality [22]. Two strong risk factors for NSCLC development—age and smoking—are concurrent risk and prognostic factors for many cardiovascular, respiratory, and metabolic conditions [30–32]. Thus, it was important to determine that the performance of logcig-years could be repeated when the outcome was focused solely on LCSS.

The major strengths of this study are the multicenter, multinational design and large sample size as well as the comparison of eight smoking metrics. Previous studies that modelled smoking metrics had limited sample sizes and often compared only one or two metrics [13,33,34]. This study collected data from 25 different studies across 22 different study groups, based in 10 different countries, to model eight continuous smoking metrics. This ensures the results of this study are broadly generalizable despite differences in smoking habits, clinical practices, and NSCLC survival between different centers/countries.

There are several limitations to this study. Firstly, ILCCO data were collected over a 36-year period beginning in 1983, and information on certain smoking exposure variables (e.g. time-to-first cigarette), secondhand smoke exposure, comorbidities, and NSCLC driver mutations were not consistently available across participating centers. Advances in NSCLC treatment guided by driver mutations have substantially improved outcomes in recent years; however, detailed treatment data were not available within the ILCCO dataset. Year-of-diagnosis was included as a covariate to partially account for improvements over time in NSCLC management and outcomes. Additionally, post-diagnosis prognostic factors such as healthcare utilization, quality of life, and changes in health behaviors were unavailable [35]. Thus, these potentially important prognostic factors could not be incorporated into adjusted models. Secondly, all smoking data from ILCCO was self-reported, without biochemical corroboration available, which is considered the gold-standard for measuring smoking exposure [36]. However, population-based epidemiological studies find self-reported smoking data to be generally accurate [37]. Moreover, reliance on self-reported smoking history may better reflect what is available in typical clinical and research settings.

Our findings suggest that more granular documentation of smoking exposure may improve prognostic modelling in NSCLC. In clinical and research documentation, we recommend routinely capturing base smoking metrics (e.g. cigarettes-per-day, smoking duration, years-since-quitting) in addition to binary smoking status or pack-years alone. Collecting these base metrics allows for a more comprehensive characterization of smoking exposure and enables calculation of alternative smoking metrics, such as logcig-years, for use in LC registries and prognostics assessment models.

There are several areas of future research. Firstly, these findings should be validated in cohorts with comprehensive molecular and treatment data to determine which smoking metrics models survival best when treatment data is available. Secondly, many existing metrics neglect potentially valuable aspects of smoking exposure (e.g. time-to-first cigarette) that may improve modelling the exposure-survival association. Furthermore, existing metrics (like logcig-years) were developed in the LC risk setting. Future studies may consider developing novel smoking metrics that incorporate additional dimensions of smoking exposure and are specifically tailored to modelling survival. Finally, our study used regression analyses and spline-based modelling to compare smoking metrics. However, machine learning approaches have shown promise in identifying important molecular targets, novel drug pathways, and even summarizing existing evidence [38,39]. Future research should evaluate whether integrating machine learning approaches can improve modelling smoking exposure beyond traditional regression-based approaches.

## 5. Conclusion

In conclusion, logcig-years and square-root pack-years most accurately captured the relationship between smoking exposure and overall survival (OS) in NSCLC patients. Both metrics demonstrated a larger effect size and had a better linear model fit compared to the conventionally used pack-years. Logcig-years also most accurately captured the relationship between smoking exposure and LCSS as well as demonstrated consistent associations across age, sex, cancer stage, and histology. These findings support logcig-years as a robust and reliable metric for quantifying smoking exposure in NSCLC patients, with applications in both LC research and clinical practice.

## Supplementary Material

Suppl2

Suppl3

Suppl1

## Figures and Tables

**Table 1:** Demographics, clinical characteristics, and smoking metrics of the study cohort.

Clinical Characteristic	Categories, n (%) unless specified	Total (n = 28,702)

**Age at Diagnosis**	Median [IQR], years	64 [57–71]
**Sex**	Males	15,144 (53)
**Race**	White	21,619 (75)
	Non-white	7083 (25)
**Education**	No high school	3733 (13)
	High school	7872 (27)
	Post-secondary	7357 (26)
	Other/missing	9740 (34)
**Body Mass Index**	Median (IQR)	25 [22–29]
**Histology**	Adenocarcinoma	17,053 (59)
	Squamous cell	6995 (24)
	Large cell/other	4654 (16)
**Clinical Stage at Diagnosis**	I	9200 (32)
	II	3434 (12)
	III	7255 (25)
	IV	8813 (31)
**Smoking Status**	Current	12,883 (45)
	Former	8652 (30)
	Never	7167 (25)
**Cigarettes per Day**	Median [IQR]	20 [0.6–27]
**Smoking Duration**	Median [IQR], years	35 [1–45]
**Pack years**	Median [IQR]	35 [0.1–54]
**Square root Pack years**	Median [IQR]	6 [0.4–7]
**Logcig-years**	Median [IQR]	107 [1–141]
**CSI**	Median [IQR]	10 [0–15]
**Age of Initiation**	Median [IQR], years	18 [15–21]
**Smoking Abstinence Duration**	Median [IQR], years	0 [0–8]

**Abbreviations:** IQR, interquartile range; CSI, comprehensive smoking index.

**Table 2:** Adjusted Cox-proportional hazards models, linear models, and non-linear models of the eight smoking metrics in relation to overall survival. Models were adjusted for smoking status, age, sex, race, education, body mass index, cancer stage, histology, and year of diagnosis. The highest significant adjusted hazard ratios and lowest AIC-linear values are bolded.

Smoking Metric	n	aHR (95% CI) per standard deviation	AIC-linear	p-linear	p-nonlinear

**Cigarettes per day**	28,702	1.08 (1.06–1.10)	263,160	< 0.001	< 0.001
**Duration**	28,702	1.10 (1.08–1.12)	263,138	< 0.001	0.01
**Pack years**	28,702	1.09 (1.07 –1.11)	263,141	< 0.001	0.001
**Square root pack years**	28,702	**1.11 (1.09–1.13)**	263,126	< 0.001	0.15
**Logcig-years**	28,702	**1.11 (1.09–1.13)**	**263,122**	< 0.001	0.002
**CSI**	28,702	1.09 (1.07–1.11)	263,139	< 0.001	0.001
**Age of initiation**	21,485	1.01 (0.99–1.03)	213,271^a^	0.31	0.43
**Years since quit**	21,485	0.96 (0.94–0.98)	213,255^a^	< 0.001	0.008

**Abbreviations:** CSI, comprehensive smoking index; aHR, adjusted hazard ratio; CI, confidence interval; AIC, Akaike information criterion.

a Difference in sample size precludes direct comparison of AIC-linear values.

**Table 3:** Adjusted Cox-proportional hazards models, linear models, and non-linear models for the three best performing smoking metrics for overall survival stratified by age, sex, smoking status, cancer stage, and histology. Pack years was also included as it is a conventionally used metric. Models were adjusted for smoking status, age, sex, race, education, body mass index, cancer stage, histology, and year of diagnosis. The highest significant aHR and lowest AIC-linear values are bolded.

Subgroup	Smoking Metric	aHR (95% CI) per standard deviation	AIC-linear	p-linear	p-nonlinear

**Age (<65 n = 14,882,≥65 n = 13,820)**					
**< 65**	**Duration**	**1.13 (1.10–1.17)**	113,046	< 0.001	0.20
	**Pack years**	1.11 (1.08–1.14)	113,055	< 0.001	0.002
	**Square root pack years**	**1.13 (1.10–1.16)**	113,044	< 0.001	0.13
	**Logcig-years**	**1.13 (1.10–1.17)**	**113,042**	< 0.001	0.13
**≥ 65**	**Duration**	1.13 (1.10–1.16)	129,376	< 0.001	0.33
	**Pack years**	1.13 (1.10–1.16)	129,372	< 0.001	< 0.001
	**Square root pack years**	**1.15 (1.12–1.18)**	129,357	< 0.001	0.70
	**Logcig-years**	**1.15 (1.12–1.18)**	**129,354**	< 0.001	0.57
**Sex (Male n = 15,144, Female n = 13,558)**					
**Male**	**Duration**	1.10 (1.07–1.13)	139,072	< 0.001	0.41
	**Pack years**	1.09 (1.06–1.11)	139,072	< 0.001	0.02
	**Square root pack years**	1.10 (1.07–1.13)	139,066	< 0.001	0.58
	**Logcig-years**	**1.11 (1.08–1.14)**	**139,059**	< 0.001	0.12
**Female**	**Duration**	1.09 (1.06–1.13)	103,966	< 0.001	0.001
	**Pack years**	1.08 (1.06–1.11)	103,969	< 0.001	< 0.001
	**Square root pack years**	**1.10 (1.07–1.13)**	**103,959**	< 0.001	0.002
	**Logcig-years**	**1.10 (1.07–1.13)**	103,962	< 0.001	0.001
**Smoking Status (Current n = 12,883, Former n = 8652)**					
**Current**	**Duration**	1.01 (0.98–1.05)	115,623	0.58	< 0.001
	**Pack years**	**1.04 (1.01–1.07)**	**115,614**	0.002	0.21
	**Square root pack years**	**1.04 (1.02–1.07)**	**115,614**	0.002	0.07
	**Logcig-years**	**1.04 (1.01–1.07)**	115,616	0.007	< 0.001
**Former**	**Duration**	1.04 (1.01–1.08)	81,435	0.02	0.004
	**Pack years**	**1.06 (1.03–1.09)**	**81,426**	< 0.001	0.09
	**Square root pack years**	1.05 (1.02–1.09)	81,430	0.001	0.003
	**Logcig-years**	**1.06 (1.03–1.10)**	81,429	0.001	0.001
**Cancer Stage (Non-metastatic n = 19,889, Metastatic n = 8813)** ^a^					
**Non-metastatic**	**Duration**	1.12 (1.10–1.15)	168,365	< 0.001	0.04
	**Pack years**	1.10 (1.07–1.12)	168,385	< 0.001	< 0.001
	**Square root pack years**	1.12 (1.10–1.15)	168,367	< 0.001	0.002
	**Logcig-years**	**1.13 (1.10–1.16)**	**168,360**	< 0.001	0.001
**Metastatic**	**Duration**	1.06 (1.03–1.10)	79,228	< 0.001	0.001
	**Pack years**	1.08 (1.05–1.11)	79,220	< 0.001	0.007
	**Square root pack years**	**1.09 (1.06–1.13)**	**79,213**	< 0.001	0.36
	**Logcig-years**	1.08 (1.05–1.12)	79,221	< 0.001	0.07
**Histology (Adenocarcinoma n = 17,053, Squamous Cell n = 6995)**					
**Adenocarcinoma**	**Duration**	1.13 (1.10–1.16)	128,532	< 0.001	0.12
	**Pack years**	1.12 (1.10–1.15)	128,527	< 0.001	0.05
	**Square root pack years**	**1.14 (1.11–1.16)**	128,521	< 0.001	0.30
	**Logcig-years**	**1.14 (1.11–1.17)**	**128,517**	< 0.001	0.04
**Squamous Cell**	**Duration**	**1.05 (1.01–1.09)**	59,969	0.02	0.06
	**Pack years**	1.04 (1.01–1.07)	59,970	0.02	0.61
	**Square root pack years**	1.04 (1.01–1.08)	59,970	0.02	0.61
	**Logcig-years**	**1.05 (1.02–1.09)**	**59,967**	0.005	0.08

**Abbreviations:** aHR, adjusted hazard ratio; CI, confidence interval; AIC, Akaike information criterion.

a Non-metastatic was defined as stages I–III, metastatic was defined as stage IV.

**Table 4:** Adjusted Cox-proportional hazards models, linear models, and non-linear models of the eight-smoking metrics in relation to lung cancer-specific survival. Models were adjusted for smoking status, age, sex, race, education, body mass index, cancer stage, histology, and year of diagnosis. The highest significant aHR and lowest AIC-linear values were bolded.

Smoking Metric	n	aHR (95% CI) per standard deviation	AIC-linear	p-linear	p-nonlinear

**Cigarettes per day**	18,957	1.06 (1.03–1.09)	113,458	< 0.001	0.10
**Duration**	18,957	1.07 (1.04–1.11)	113,454	< 0.001	0.14
**Pack years**	18,957	1.08 (1.05–1.11)	113,447	< 0.001	0.43
**Square root pack years**	18,957	1.08 (1.05–1.11)	113,449	< 0.001	0.09
**Logcig-years**	18,957	**1.09 (1.05–1.12)**	**113,443**	< 0.001	0.002
**CSI**	18,957	1.08 (1.05–1.11)	113,447	< 0.001	0.53
**Age of initiation**	13,871	1.03 (1.00–1.06)	92,457	0.09	0.23
**Years since quit**	13,871	0.94 (0.92–0.97)	92,443	< 0.001	0.04

**Abbreviations:** CSI, comprehensive smoking index; aHR, adjusted hazard ratio; CI, confidence interval; AIC, Akaike information criterion.

## Data Availability

The data underlying this article was provided by the International Lung Cancer Consortium with permission. Data will be shared on request to the corresponding author with permission of the International Lung Cancer Consortium.

## Appendix A. Supporting information

Supplementary data associated with this article can be found in the online version at doi:10.1016/j.canep.2026.103052.

## Glossary

AIC: Akaike Information Criterion
AIC-linear: Akaike Information Criterion assuming a linear relationship
BMI: Body Mass Index
CONSORT: Consolidated Standards of Reporting Trials
CSI: Comprehensive Smoking Index
HR: Hazard Ratio
ILCCO: International Lung Cancer Consortium
IQR: Interquartile Range
KM: Kaplan–Meier
LC: Lung Cancer
LCSS: Lung Cancer–Specific Survival
NSCLC: Non–Small Cell Lung Cancer
OS: Overall Survival
TNM: Tumor, Node, Metastasis Staging System
