# Supplementary material for: Evaluating eight smoking metrics for modelling survival in non-small cell lung cancer
Source: Cancer Epidemiol. Author manuscript; Available in PMC 2026 Apr 29. (PMC13123461; doi:10.1016/j.canep.2026.103052)
Supplement: Suppl2 [file NIHMS2162957-supplement-Suppl2.docx]

**SUPPLEMENTARY MATERIAL**

**Supplementary Material 1: Data Collection, Harmonization, and Management**

In total, 25 studies were included in this study, 15 from North America, six from Europe, three from Asia, and one from South America. Smoking information and other demographic details were collected once at the time of patient recruitment into the study. Twenty-three studies were case-control studies. From these studies, only the cases were included in our analysis and smoking exposure was assessed at the time of lung cancer diagnosis. Two studies were cohort studies. From these studies, smoking was assessed at the time of study enrollment, which may predate lung cancer diagnosis. The exact inclusion and exclusion criteria for studies into the International Lung Cancer Consortium can be found on the website (<http://ilcco.iarc.fr>). For the analysis presented in this study, patients were required to be ≥18-year-old, had histologically confirmed NSCLC, have complete demographic, clinical (including histology, date-of-diagnosis, stage-at-diagnosis, vital status, and date of last follow-up and/or death), and smoking information (including smoking status, cigarettes-per-day, smoking duration, age-of-initiation, and years-since-quit) for inclusion. Data across studies were coded and harmonized into a single dataset for this study.

To account for behavioral changes that may transiently occur due to illness and/or the new cancer diagnosis, the definition of a former smoker is a patient who has quit smoking for ≥two years, and a current smoker includes patients who have smoked as recently as two years. Although different smoking questionnaires were used between the different studies, inclusion into the International Lung Cancer Consortium required specific data fields to be collected by all studies. This allowed for harmonization between studies.  Data from each center was examined for missing data and abnormal values. These discrepancies were sent to the individual principal investigator for each study who provided updated data and resolved discrepancies. Subjects with missing demographic data (including age and sex), smoking metrics, and clinical data (including lung cancer stage, year of diagnosis, and survival status) were excluded.

**Supplementary Material 2**

**Pack years formula:**

$$\left( {packs of cigarettes}/{day} \right)x years smoked$$

One pack of cigarettes is defined as 20 cigarettes.

**Square-root pack years formula:**

$$\surd[\left( {packs of cigarettes}/{day} \right)x years smoked]$$

**Log-cig years formula:**

$$\log\left( cigarettes/day+1 \right)x years smoked$$

**Comprehensive Smoking Index (CSI) formula:**

$$\left( 1-{0.5}^{\frac{duration}{\tau}} \right)\left( {0.5}^{\frac{years since quit}{\tau}} \right)(cigarettes/day)$$

*τ* is a half-life parameter measured in years determined by maximizing the model fit for our specific dataset in a process outlined by Papadopoulos et al.[40]

**SUPPLEMENTARY FIGURE CAPTIONS**

**Supplementary Figure 1.** CONSORT (Consolidated Standards of Reporting Trials) diagram of participant inclusion and exclusion within this International Lung Cancer Consortium study.

**Supplementary Figure 2.** Unadjusted Kaplan Meier overall survival curves presented for each smoking metric. Continuous smoking metrics are divided into four quantiles with cut-offs at 25%, 50%, and 75% as well as a separate line for never smokers. The figure includes the log-rank test p-values.

**Supplementary Figure 3.** Spline-curves for the three best performing smoking metrics (smoking duration, square root pack years, and logcig-years) and pack years in relation to overall survival. Splines are stratified by age, sex, smoking status, cancer stage, and histology. Splines are adjusted for smoking status, age, sex, race, education, body mass index, cancer stage, histology, and year of diagnosis.

**Supplementary Figure 4.** Spline-curves for the three best performing smoking metrics (smoking duration, square root pack years, and logcig-years) and pack years in relation to overall survival. Splines are stratified by individual cancer stages. Splines are adjusted for smoking status, age, sex, race, education, body mass index, histology, and year of diagnosis.

**Supplementary Figure 5.** Spline-curves for eight smoking metrics in relation to lung cancer-specific survival adjusted for smoking status, age, sex, race, education, body mass index, cancer stage, histology, and year of diagnosis. Note that age of initiation and years since quit have a different sample size than the other smoking metrics. Abbreviations: CSI, comprehensive smoking index.
