# Supplementary material for: Evaluating eight smoking metrics for modelling survival in non-small cell lung cancer
Source: Cancer Epidemiol. Author manuscript; Available in PMC 2026 Apr 29. (PMC13123461; doi:10.1016/j.canep.2026.103052)
Supplement: Suppl3 [file NIHMS2162957-supplement-Suppl3.docx]

**SUPPLEMENTARY TABLES**

| **Study name** | **Region** | **Study Design** | **Enrollment period** | **Patients assessed for eligibility n (%)** | **Patients included**  **n (%)** | **Current smokers  n (%)** | **Former smokers**  **n (%)** | **Never smokers**  **n (%)** | **Median Follow-up Years** |
| --- | --- | --- | --- | --- | --- | --- | --- | --- | --- |
| **Mayo-EGLC** | USA | Case-control | 1984–2016 | 13,441 | 10,153 (35) | 4,003 (31) | 3,974 (46) | 2,176 (30) | 12 |
| **LCS** | USA | Case-control | 1983–2010 | 3,506 | 2,615 (9) | 1,011 (8) | 1,165 (13) | 439 (6) | 8.6 |
| **LUN MLT** | Europe & Russia | Case-control | 2005–2016 | 2,262 | 2,145 (7) | 1,323 (10) | 447 (5) | 375 (5) | 4.3 |
| **MSH-PMH** | Canada | Case-control | 2007–2013 | 2,565 | 2,110 (7) | 783 (6) | 645 (7) | 682 (10) | 6.5 |
| **FUDAN** | China | Case-control | 2009–2014 | 1,721 | 1,699 (6) | 596 (5) | 143 (2) | 960 (13) | 2.9 |
| **NCCRI-JAPAN** | Japan | Case-control | 2000–2016 | 1,494 | 1,437 (5) | 472 (4) | 214 (2) | 751 (10) | 5.0 |
| **EAGLE** | Europe | Case-control | 2002–2005 | 1,442 | 1,286 (4) | 739 (6) | 425 (5) | 122 (2) | 7.0 |
| **NCI-MD** | USA | Case-control | 1998–2015 | 1,357 | 1,104 (4) | 581 (5) | 403 (5) | 120 (2) | 9.2 |
| **NANJING** | China | Case-control | 2002–2010 | 860 | 849 (3) | 468 (4) | 0 (0) | 381 (5) | 4.3 |
| **CARET** | USA | Cohort | 1985–1994 | 763 | 756 (3) | 601 (5) | 153 (2) | 2 (0) | 14 |
| **EXHALE** | USA | Case-control | 2005–2010 | 639 | 569 (2) | 331 (3) | 205 (2) | 33 (0) | 8.3 |
| **WELD** | USA | Case-control | 2001–2005 | 565 | 513 (2) | 290 (2) | 170 (2) | 53 (1) | 12 |
| **INHALE** | USA | Case-control | 2011–2018 | 465 | 393 (1) | 238 (2) | 112 (1) | 43 (1) | 2.0 |
| **DCC** | USA | Case-control | 1991–1997 | 436 | 384 (1) | 201 (2) | 146 (2) | 37 (1) | 21 |
| **ACS-CPSII** | USA | Cohort | 1992–2006 | 427 | 357 (1) | 138 (1) | 170 (2) | 49 (1) | 17 |
| **CAPUA** | Spain | Case-control | 2002–2012 | 408 | 353 (1) | 305 (2) | 0 (0) | 48 (1) | 14 |
| **MDACC_LC** | USA | Case-control | 1991–2016 | 348 | 334 (1) | 260 (2) | 0 (0) | 74 (1) | 2.6 |
| **LCRINS** | Spain | Case-control | 2006–2016 | 328 | 328 (1) | 0 (0) | 0 (0) | 328 (5) | 5.5 |
| **LLP** | England | Case-control | 1996–2016 | 317 | 265 (1) | 106 (1) | 99 (1) | 60 (1) | 7.0 |
| **FHSIII** | USA | Case-control | 1990–2003 | 246 | 240 (1) | 192 (1) | 26 (0) | 22 (0) | 14 |
| **Barretos-Brazil** | Brazil | Case-control | 2010–2020 | 614 | 234 (1) | 0 (0) | 0 (0) | 234 (3) | 4.9 |
| **ReSoluCent** | England | Case-control | 2001–2013 | 235 | 219 (1) | 115 (1) | 68 (1) | 36 (1) | 6.1 |
| **NELCS** | USA | Case-control | 2005–2007 | 215 | 190 (1) | 119 (1) | 63 (1) | 8 (0) | 8.0 |
| **TLC** | USA | Case-control | 2001–2013 | 679 | 124 (0) | 0 (0) | 0 (0) | 124 (2) | 2.9 |
| **ESTHER** | Germany | Case-control | 2000–2004 | 63 | 45 (0) | 11 (0) | 24 (0) | 10 (0) | 13 |
| **Total** |  |  |  | **35,396** | **28,702** | **12,883** | **8,652** | **7,167** |  |

**Supplementary Table 1.** Number and characteristics of participants are stratified by International Lung Cancer Consortium study site.

**Abbreviations:** Mayo-EGLC, Epidemiology & Genetics of Lung Cancer (Mayo Clinic); USA, United States of America; LCS, Harvard Lung Cancer Study; LUN MLT, Early stage non-small cell lung cancer study, Russian Genetic Epidemiology Study of Multiple Cancer Sites (International Agency for Research on Cancer); MSH-PMH, Multi-Cancer Case-Control Thoracic Study (Sinai Health System, Princess Margaret Cancer Centre); FUDAN, Fudan University (Shanghai); NCCRI-JAPAN, National Cancer Center Research Institute (Japan); EAGLE, Environment And Genetics in Lung cancer Etiology (National Cancer Institute, US National Institutes of Health); NCI-MD, National Cancer Institute-MD Case Control Study (US National Institutes of Health); NANJING, Nanjing dataset (Nanjing Medical University School of Public Health); CARET, beta-Carotene And Retinol Efficacy Trial (Fred Hutchinson Cancer Center); TLC, Total Lung Care (Moffit Cancer Center); Barretos-Brazil (Molecular Oncology Research Center, Barretos Cancer Hospital, Barretos, Brazil); EXHALE, Exploring Health, Ancestry and Lung Epidemiology (Karmanos Cancer Institute, Wayne State University); WELD, Women’s Epidemiology of Lung Disease (Karmanos Cancer Institute, Wayne State University); INHALE, Inflammation, Health, and Lung Epidemiology (Karmanos Cancer Institute, Wayne State University); ACS-CPSII; American Cancer Society-Cancer Prevention Study II; DCC, Diet and Cancer Study (University of Hawaii Cancer Center); CAPUA: Cancer de Pulmón en Asturias (Universidad de Oviedo, Spain); MDACC_LC, MD Anderson-Cancer Center_Lung Cancer Study; LCRINS, Lung Cancer in Never smokers (Universidad de Santiago de Compostela, Spain); LLP, Liverpool Lung Project (University of Liverpool); FHSIII, Family Health Study III (Karmanos Cancer Institute); ReSoluCent, Resource for the Study of Lung Cancer Epidemiology in North Trent (National Institute for Health Research, University of Sheffield); NELCS, New England Lung Cancer Study (Geisel School of Medicine at Dartmouth); ESTHER, Epidemiological study on chances of prevention, early detection and optimized therapy of chronic diseases in the older population (German Cancer-research center, Department of Clinical Epidemiology and Aging Research). (Barretos, Brazil, Barretos Cancer Hospital, Brazil).

**Supplementary Table 2.** Unadjusted Cox-proportional hazards models, linear models, and non-linear models of the eight smoking metrics in relation to overall survival and lung cancer-specific survival. Models are still adjusted for smoking status as none of the smoking metrics except the comprehensive smoking index account for smoking status. The highest significant aHR and lowest AIC-linear values are bolded.

| **Smoking Metric** | | **n** | **HR (95% CI) per  standard deviation** |  | **AIC-linear** | **p-linear** | **p-nonlinear** |
| --- | --- | --- | --- | --- | --- | --- | --- |
|  | **Overall Survival** | | | | | | |
| **Cigarettes per day** | | 28,702 | 1.12 (1.11–1.14) |  | 376,542 | <0.001 | <0.001 |
| **Duration** | | 28,702 | 1.16 (1.15–1.18) |  | 376,388 | <0.001 | <0.001 |
| **Pack years** | | 28,702 | 1.15 (1.13–1.16) |  | 376,426 | <0.001 | <0.001 |
| **Square root pack years** | | 28,702 | 1.16 (1.14–1.18) |  | 376,399 | <0.001 | 0.11 |
| **Logcig-years** | | 28,702 | **1.17 (1.15–1.19)** |  | **376,353** | <0.001 | <0.001 |
| **CSI** | | 28,702 | 1.13 (1.12–1.15) |  | 376,484 | <0.001 | <0.001 |
| **Age of initiation** | | 21,485 | 1.00 (0.99–1.02) |  | 291,404^a^ | 0.82 | 0.02 |
| **Years since quit** | | 21,485 | 1.00 (0.98–1.01) |  | 291,404^a^ | 0.64 | 0.01 |
|  | **Lung Cancer-specific Survival** | | | | | | |
| **Cigarettes per day** | | 18,957 | 1.15 (1.13–1.17) |  | 158,750 | <0.001 | <0.001 |
| **Duration** | | 18,957 | **1.24 (1.21–1.27)** |  | **158,564** | <0.001 | 0.007 |
| **Pack years** | | 18,957 | 1.19 (1.17–1.22) |  | 158,653 | <0.001 | <0.001 |
| **Square root pack years** | | 18,957 | 1.22 (1.19–1.24) |  | 158,616 | <0.001 | 0.05 |
| **Logcig-years** | | 18,957 | **1.24 (1.21–1.26)** |  | 158,566 | <0.001 | 0.001 |
| **CSI** | | 18,957 | 1.20 (1.17–1.21) |  | 158,661 | <0.001 | <0.001 |
| **Age of initiation** | | 13,871 | 0.96 (0.94–0.99) |  | 122,618^a^ | 0.004 | 0.01 |
| **Years since quit** | | 13,871 | 0.93 (0.91–0.96) |  | 122,596^a^ | <0.001 | 0.003 |

**Abbreviations:** HR, hazard ratio; CI, confidence interval; AIC, Akaike information criterion; CSI, comprehensive smoking index.
^a^ Difference in sample size precludes direct comparison of AIC-linear values.

**Supplementary Table 3.** Adjusted Cox-proportional hazards models, linear models, and non-linear models for the three best performing smoking metrics for overall survival stratified by individual cancer stage. Pack years was also included as it is a conventionally used metric. Models were adjusted for smoking status, age, sex, race, education, body mass index, histology, and year of diagnosis. The highest significant aHR and lowest AIC-linear values are bolded.

| **Subgroup (n)** | **Smoking Metric** | **aHR (95% CI) per standard deviation** |  | **AIC-linear** | **p-linear** | **p-nonlinear** |
| --- | --- | --- | --- | --- | --- | --- |
| **Stage I** | **Duration** | 1.30 (1.24–1.36) |  | 56,489 | <0.001 | 0.037 |
| **(n=9,200)** | **Pack years** | 1.20 (1.16–1.25) |  | 56,536 | <0.001 | <0.001 |
|  | **Square root pack years** | 1.28 (1.23–1.34) |  | 56,499 | <0.001 | 0.008 |
|  | **Logcig-years** | **1.31 (1.25–1.36)** |  | **56,481** | <0.001 | 0.078 |
| **Stage II** | **Duration** | **1.07 (1.01–1.13)** |  | **24,970** | 0.023 | 0.16 |
| **(n=3,434)** | **Pack years** | 1.05 (1.00–1.10) |  | 24,972 | 0.065 | 0.61 |
|  | **Square root pack years** | 1.05 (1.00–1.12) |  | 24,972 | 0.066 | 0.58 |
|  | **Logcig-years** | **1.07 (1.01–1.13)** |  | **24,970** | 0.023 | 0.21 |
| **Stage III** | **Duration** | 1.04 (1.00–1.07) |  | 65,342 | 0.063 | 0.31 |
| **(n=7,255)** | **Pack years** | **1.05 (1.02–1.09)** |  | **65,337** | 0.004 | 0.013 |
|  | **Square root pack years** | **1.05 (1.01–1.09)** |  | 65,338 | 0.009 | 0.002 |
|  | **Logcig-years** | 1.04 (1.01–1.08) |  | 65,340 | 0.027 | 0.002 |
| **Stage IV** | **Duration** | 1.06 (1.03–1.10) |  | 79,228 | <0.001 | 0.001 |
| **(n=8,813)** | **Pack years** | 1.08 (1.05–1.11) |  | 79,220 | <0.001 | 0.007 |
|  | **Square root pack years** | **1.09 (1.06–1.13)** |  | **79,213** | <0.001 | 0.36 |
|  | **Logcig-years** | 1.08 (1.05–1.12) |  | 79,220 | <0.001 | 0.068 |

**Abbreviations:** aHR, adjusted hazard ratio; CI, confidence interval; AIC, Akaike information criterion
