# Supplementary figures and images for: Evaluating eight smoking metrics for modelling survival in non-small cell lung cancer
Source: Cancer Epidemiol. Author manuscript; Available in PMC 2026 Apr 29. (PMC13123461; doi:10.1016/j.canep.2026.103052)

Figure S1

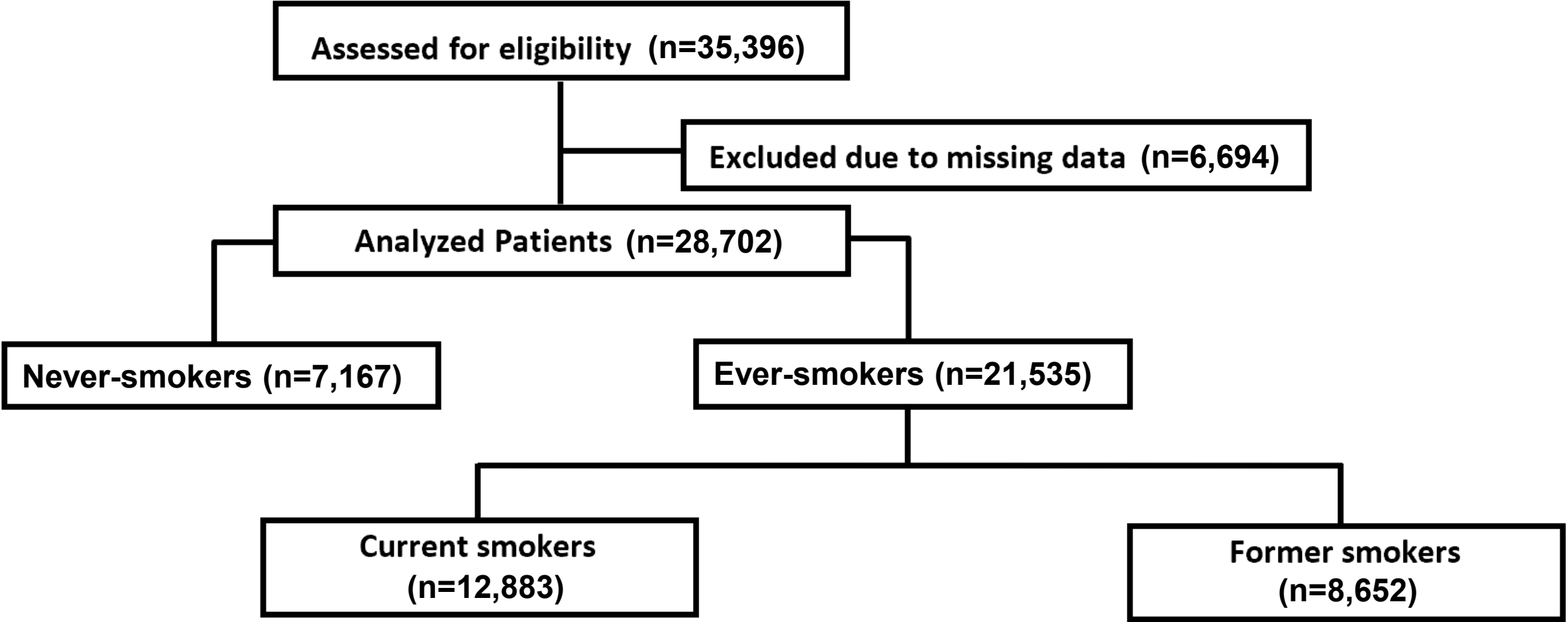

Figure S2

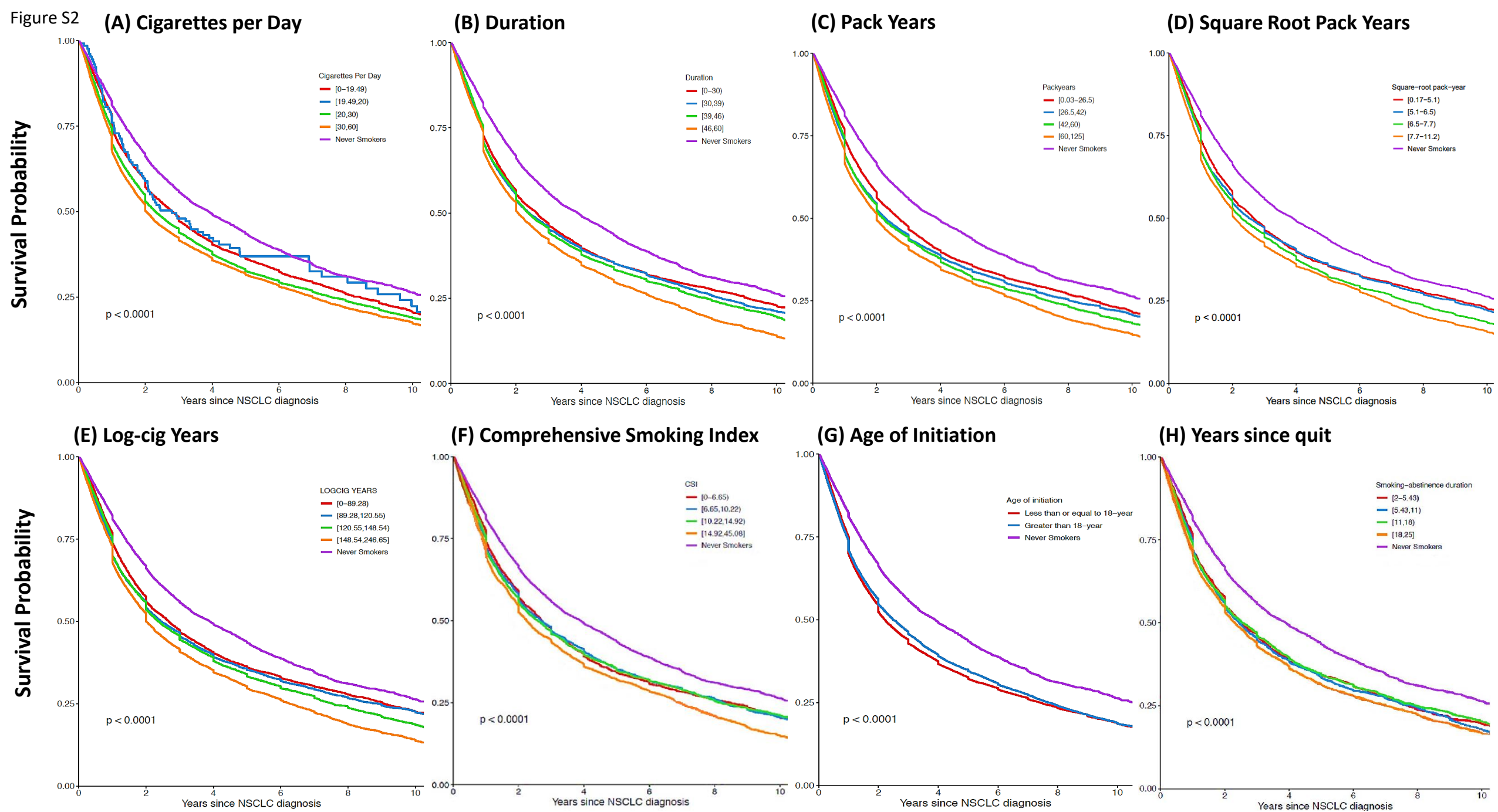

Figure S3

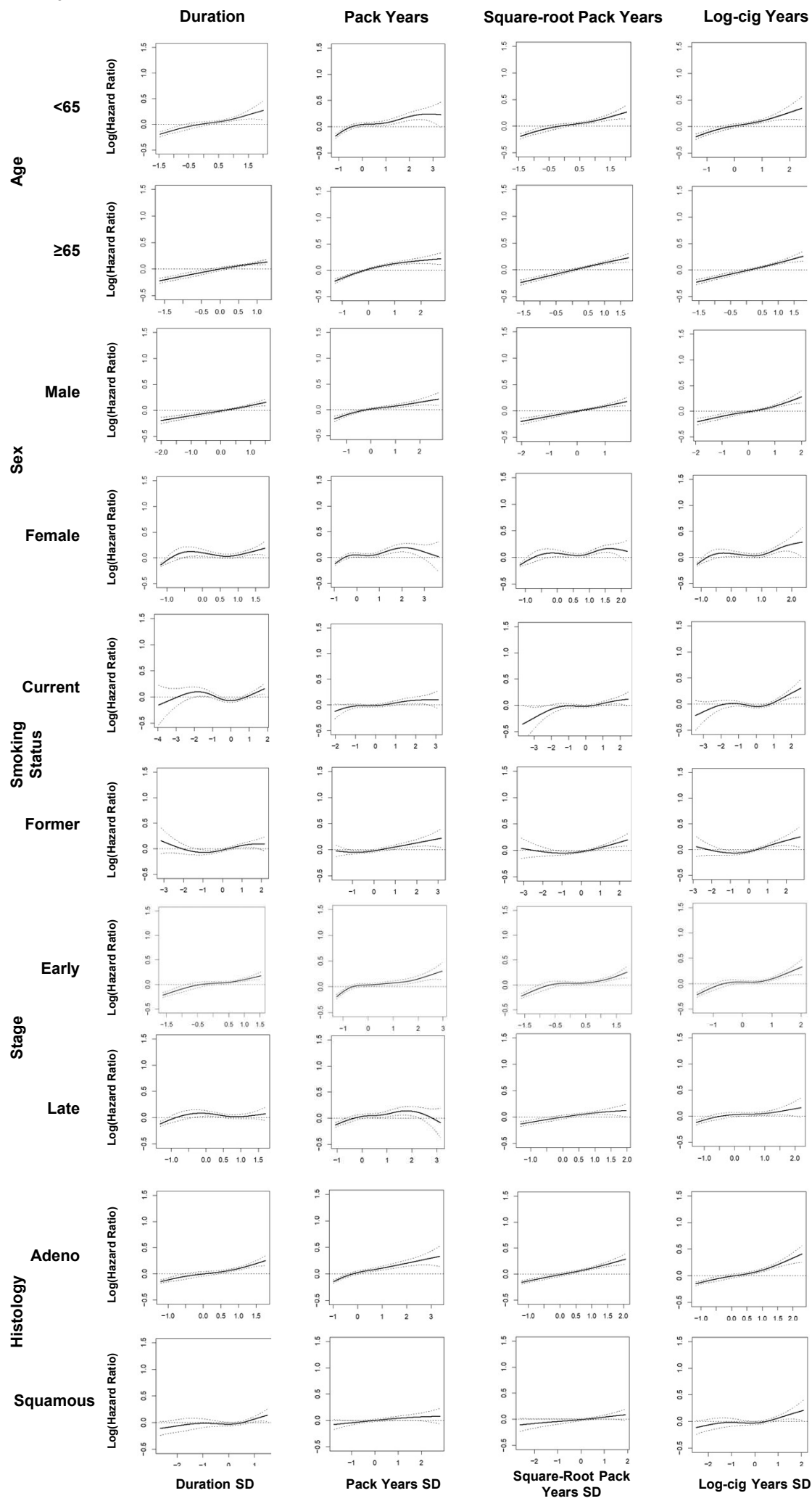

Figure S4

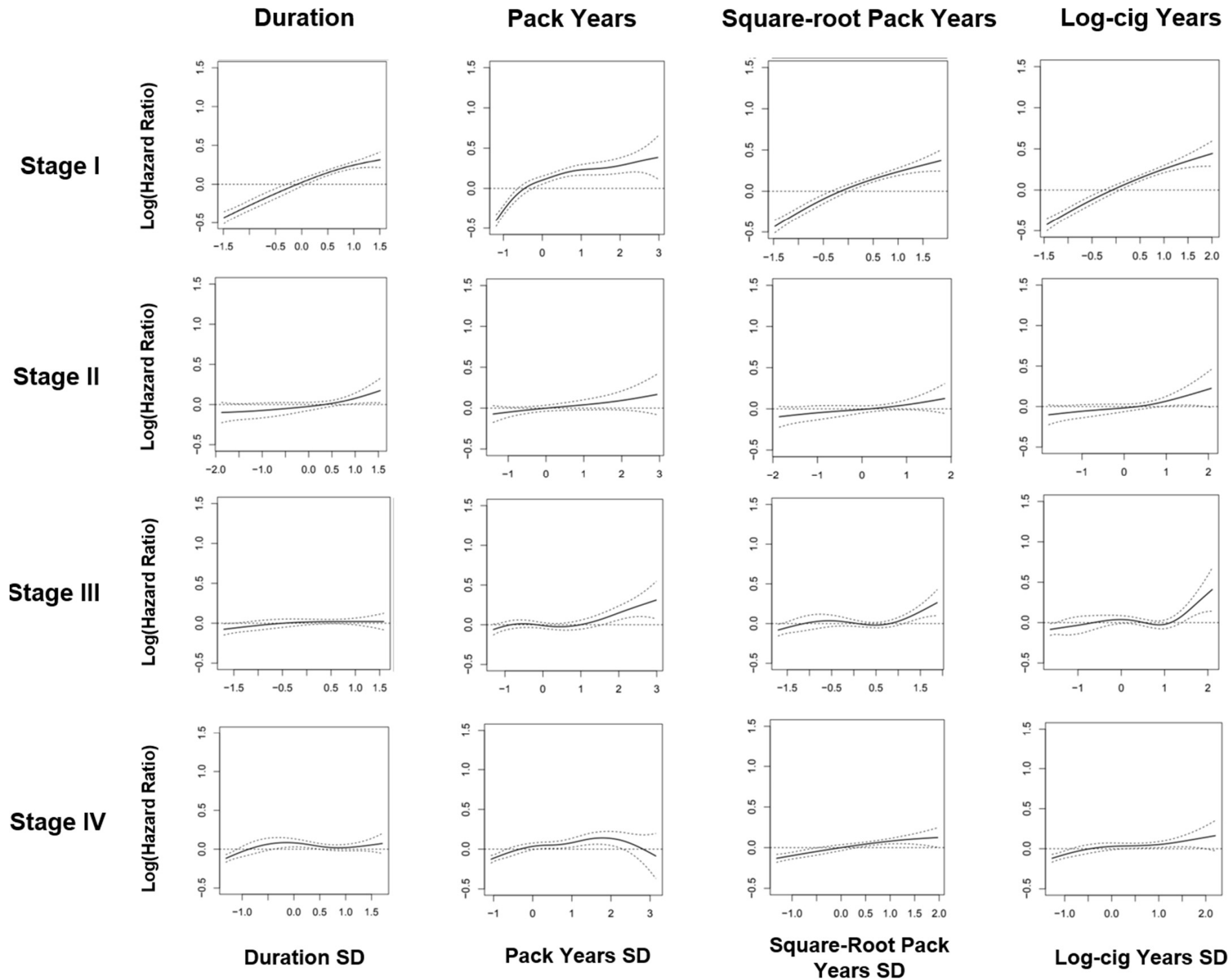

Figure S5

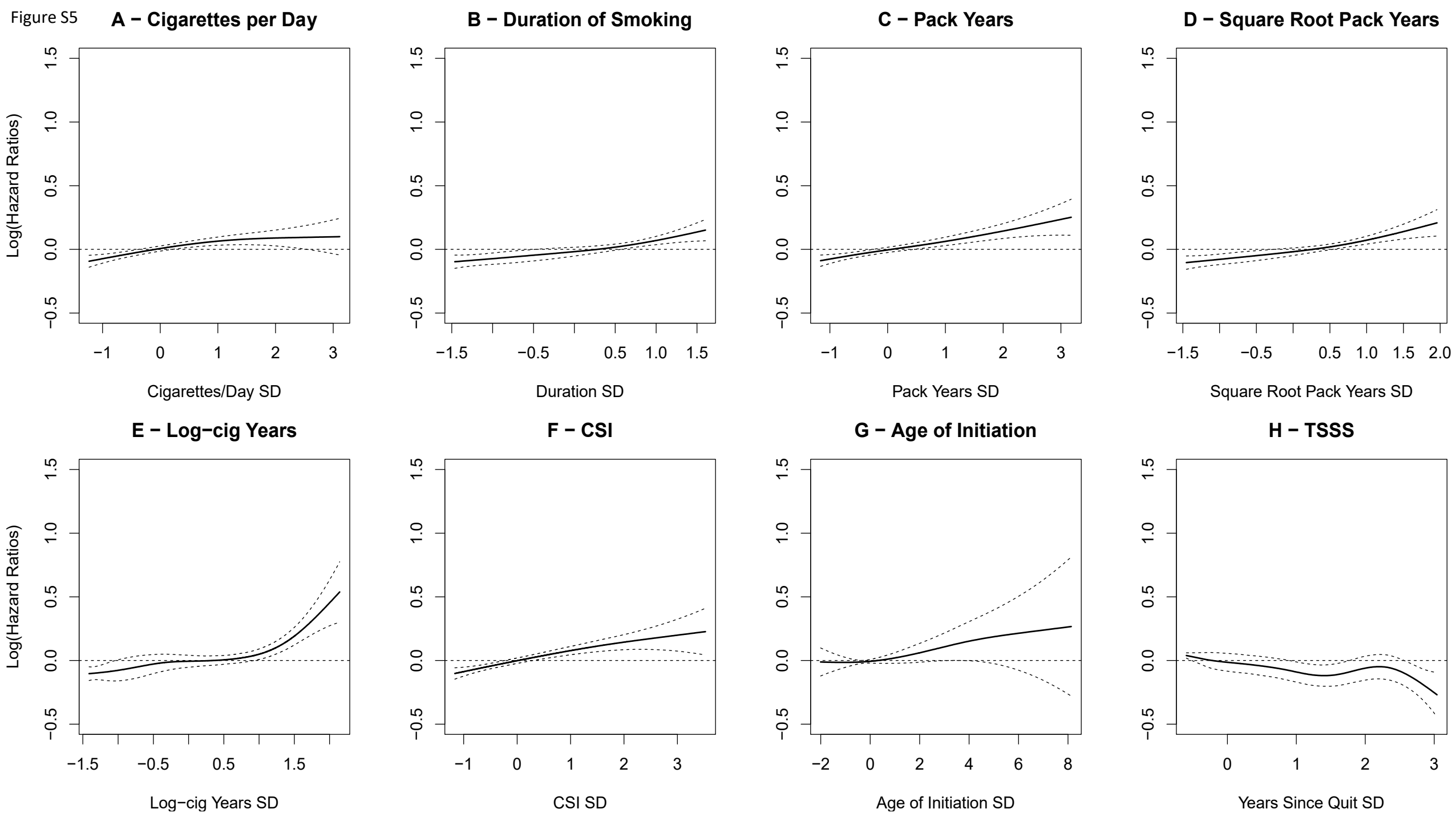

Supplement: Suppl1 [file NIHMS2162957-supplement-Suppl1.pdf]
